# 

**Published:** 1907-01

**Authors:** 


### CONTENTS.

ORIGINAL COMMUNICATIONS—

When May we Advise Gonnorrheal patients to Marry ? By W, L. Champion, M. D., Atlanta, Ga ........ 649
Address of the Retiring President, By E. Bates Block, M. D.. Atlanta, Ga ........ 652
President's Inaugural Address. By E. Bates Block M. D., Atlanta, Ga ........ 655
Cardiac Stimulation for Suspended Animation by Direct Digital Manipulation—Illustrated by Diagrams and Case Reports. By Benjamin Merrill Ricketts, M. D.. LL. D.. Cincinnati, O ........ 660

EDITORIAL NOTES AND COMMENTS—
A Criticism ........ 664
Newspaper Advertising ........ 665
NEWS AND NOTES ........ 668
BOOK REVIEWS ........ 683

SELECTIONS AND ABSTRACTS—
A German Clergyman on Quacks ........ 686
Longevity ........ 686
Ventrofixation of Uterus ........ 687
Intcstinal Obstruction ........ 688
Abscess of the Brain ........ 690
The Action of Quinine Upon the Tertian, Quartian and Estivo-Autumnal Malarial Plasmodia ........ 692
When is "Early in Operating for Appendicitis? ........ 693
"Dowieism and Eddyism" ........ 695
The Radical Cure of Aneurism ........ 697
Three Ureters Demonstrated During Lfie; Ureter-Catheterization Giving Three Difierent Urines, One Infected with Gonococci ........ 699
The Case of Dr. Matthews ........ 701
After-Care Problems ........ 703
Chronic Headache Associated with Pelvic Disease ........ 706
Treatment of Sleeplessness ........ 708
The Postal Service and the Medical Press, with reflections ........ 711

OBITUARIES—
Dr- Dharles Davis Hurt ........ 714
Dr, Hunter Pope Cooper ........ 715

